# In Situ Light‐Modulation of Capacity and Impedance in Lithium‐Ion Batteries

**DOI:** 10.1002/advs.202503340

**Published:** 2025-06-05

**Authors:** Hong Yin, Xiangxiang Yu, Yucan Zhu, Zhaohui Hou, Joao Cunha, Zhenxing Liang, Zhipeng Yu

**Affiliations:** ^1^ Key Laboratory of Hunan Province for Advanced Carbon‐based Functional Materials Hunan Institute of Science and Technology Yueyang 414006 China; ^2^ International Iberian Nanotechnology Laboratory (INL) Braga 4715‐330 Portugal; ^3^ School of Integrated Circuits Huazhong University of Science and Technology Wuhan 430074 China; ^4^ Guangdong Provincial Key Laboratory of Fuel Cell Technology School of Chemistry and Chemical Engineering South China University of Technology Guangzhou 510641 China

**Keywords:** capacities and impedances, energy storage innovation, In situ regulation, lithium storage properties, photoconductive CdS/rGO

## Abstract

The in situ regulation of capacity and impedance presents a significant challenge that impedes the application of lithium‐ion batteries (LIBs). Herein, a novel strategy is introduced that utilizes a broadband light‐modulated method for in situ manipulation of cell capacities and impedances. This approach leverages a photoconductive heterojunction comprising cadmium sulfide (CdS) nanorod arrays and a reduced graphene oxide (rGO) film. The heterostructure efficiently responds to a broad light spectrum, including UV to visible wavelengths. The results show that for the CdS/rGO anode, under conditions of UV exposure and absence of illumination, the capacity varies between 275 and 450 mAh g^−1^ after 200 cycles at 0.2 A g^−1^, and the impedance changes from 1205 to 261 Ω, respectively. When applied to a full‐cell, the capacity and impedance of the full‐cell can still be controlled by light intensity and light type. The facts suggest that by constructing light‐modulated devices, in situ modulation of battery capacity and impedance can be successfully achieved, facilitating the application of LIBs in complex scenarios. This important innovation offers a novel approach to battery design and holds immense potential for developing safer and more efficient energy storage systems.

## Graphical abstract

This study introduces a dynamic light‐controlled modulation (DLCM) anode for LIBs using CdS alignments and light‐sensitive graphene, effective across UV to visible light. The developed DLCM anode exhibits a remarkable maximum capacity of 430 mAh g^−1^ when subjected to UV light illumination, surpassing the capacity observed in the absence of illumination, which is measured at 275 mAh g^−1^ under a current density of 0.2 A g^−1^ after 200 cycles.

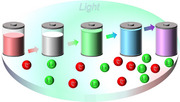

## 1 Introduction

Rechargeable lithium‐ion batteries (LIBs) have revolutionized energy storage, offering unparalleled benefits in terms of energy density and cycle life.^[^ ^1^ ^]^ Pivotal in a myriad of applications, from powering portable electronics to driving electric vehicles (EVs), LIBs have become indispensable in our technologically advanced society.^[^ ^2^ ^]^ At their core, LIBs rely on reversible redox reactions for energy storage, with the performance largely dictated by the nature of the electrode materials. These materials, critical in determining the battery's capacity, conductivity, and charge‐discharge rates, have been the subject of extensive research and development.^[^ ^3^ ^]^ Yet, despite their widespread adoption, LIBs exhibit a notable limitation. In a battery pack composed of multiple individual LIB cells, it is crucial to ensure uniformity in the impedance and capacity of each cell. However, from the moment a cell is assembled, its capacity and impedance are fixed and can only be sorted through specialized machinery.^[^ ^4^ ^]^ Cells with identical capacity and impedance can then be assembled into a battery pack. After certain cycle durations, the capacity and impedance of the cells in a battery pack will change. Deviations in these parameters can lead to uneven load distribution, accelerated degradation, and safety risks, including thermal runaway and explosions.^[^ ^5^ ^]^ Therefore, the in situ adjustment of impedance and capacity in individual cells becomes critically important. However, research in this area is almost non‐existent.

Photoconductive materials, such as germanium and zinc oxide, are a special class of semiconductors whose electrical conductivity changes with light intensity. In the dark, these materials exhibit lower conductivity, but under light exposure, the absorbed photons excites electrons from the valence band to the conduction band, generating free electrons and holes, thus increasing conductivity.^[^ ^6^ ^]^ Due to their high sensitivity to light intensity, photoconductive materials are widely used in fields such as photodetectors, solar cells, optoelectronic switches, and automatic exposure systems, playing a key role in modern optoelectronic technology.^[^ ^7^ ^]^ Certain photoconductive materials are notably used in energy storage, offering two major benefits: firstly, they enable modulation of electrode conductivity with light exposure; second, they facilitate electron and ion acceleration through an internal electric field generated in the semiconductor when illuminated. These synergistic effects collectively enhance the devices' electrochemical performance, including reduced impedance and increased capacity. At present, research into the light‐modulated performance of energy storage devices is largely undeveloped. This void offers a distinct opportunity for innovation, especially by leveraging the synergistic properties of LIBs and photoconductive materials, a new class of energy storage systems could be developed. We propose a novel concept: an in situ light‐modulated lithium‐ion battery (LM‐LIB). This integrated system is designed not only to store energy but also to dynamically adjust its capacity and impedance in response to light, potentially enhancing efficiency and adaptability in a wide range of applications, including EVs and energy storage power stations.

The key to realizing this integration lies in the development of materials that possess both high energy storage capacity and light sensitivity. Transition metal chalcogenides (TMCs) such as ZnO, SnO_2_, and CdS have emerged as promising candidates due to their ease of preparation, high theoretical capacity, and rapid photoresponse.^[^ ^8^ ^]^ However, TMC‐based anodes face significant challenges, including the continuous formation and collapse of the solid electrolyte interface (SEI), which leads to capacity degradation and compromises cycling performance.^[^ ^9^ ^]^ Furthermore, inherent drawbacks like poor conductivity and sluggish ion diffusion severely limit their cycling performance. To address these issues, incorporating carbon‐based materials onto TMC anodes, exemplified by structures like SnO_2_/graphene and ZnO/carbon dots, has been substantiated as a practical approach for enhancing conductivity, ion diffusion, and cycling stability. In addition, it should be noted that mono‐component TMC photoconductive materials are limited to responding to specific wavelength due to their inherent band gaps. For instance, ZnO, SnO_2_, and CdS exhibit bandgaps of 3.3, 3.6, and 2.4 eV, respectively. These values indicate their sensitivity for the detection of short wavelengths. Constructing heterojunctions with different semiconductor materials is an effective solution to overcome these limitations. The built‐in field of these heterojunctions enables the separation of photo‐generated carriers, thereby contributing to a swift response speed.^[^ ^10^ ^]^

Reduced graphene oxide (rGO), a well‐known p‐type semiconductor material, is characterized by its narrow bandgap, low cost, and easy preparation. The band gap and work function of rGO can be readily adjusted by controlling the reduction degree and film thickness of the material, respectively.^[^ ^11^ ^]^ Previous research has demonstrated that rGO can form a Schottky junction with semiconductors such as CdS and ZnO, resulting in a notable improvement in response speed and detection range.^[^ ^12^ ^]^ Furthermore, these heterojunctions are crucial in enhancing the conductivity and ion diffusion of TMC‐based anode materials.^[^ ^13^ ^]^ In the context of LIBs, the modulation of electronic conductivity in CdS holds significant relevance, as it exhibits desirable photoconductive properties that facilitate efficient electronic transportation during the charge and discharge processes.

In this report, we present a groundbreaking research initiative introducing a novel energy storage device termed a broadband LM‐LIB with in situ manipulated capacity and impedance. LM‐LIB combines high photoconductive CdS and rGO film with a robust heterojunction interface. This synergistic CdS/rGO composite anode exhibits remarkable properties, including efficient electron‐hole separation, controllable impedance, and enhanced Li‐ion diffusion within the LIBs, enabling rapid response to constant illumination. The introduction of light‐modulated impedance and capability in the battery device potentially constitutes a groundbreaking approach poised to optimize battery pack technology, particularly in consumer applications and battery‐only electric vehicles. The seamless integration of light‐responsive materials with conventional battery systems presents an exciting frontier for developing cutting‐edge and high‐performance energy storage solutions.

## 2 Results and Discussion

### 2.1 Broadband Light‐Modulated Anode Resign and Structural Characterization

**Figure 1:**
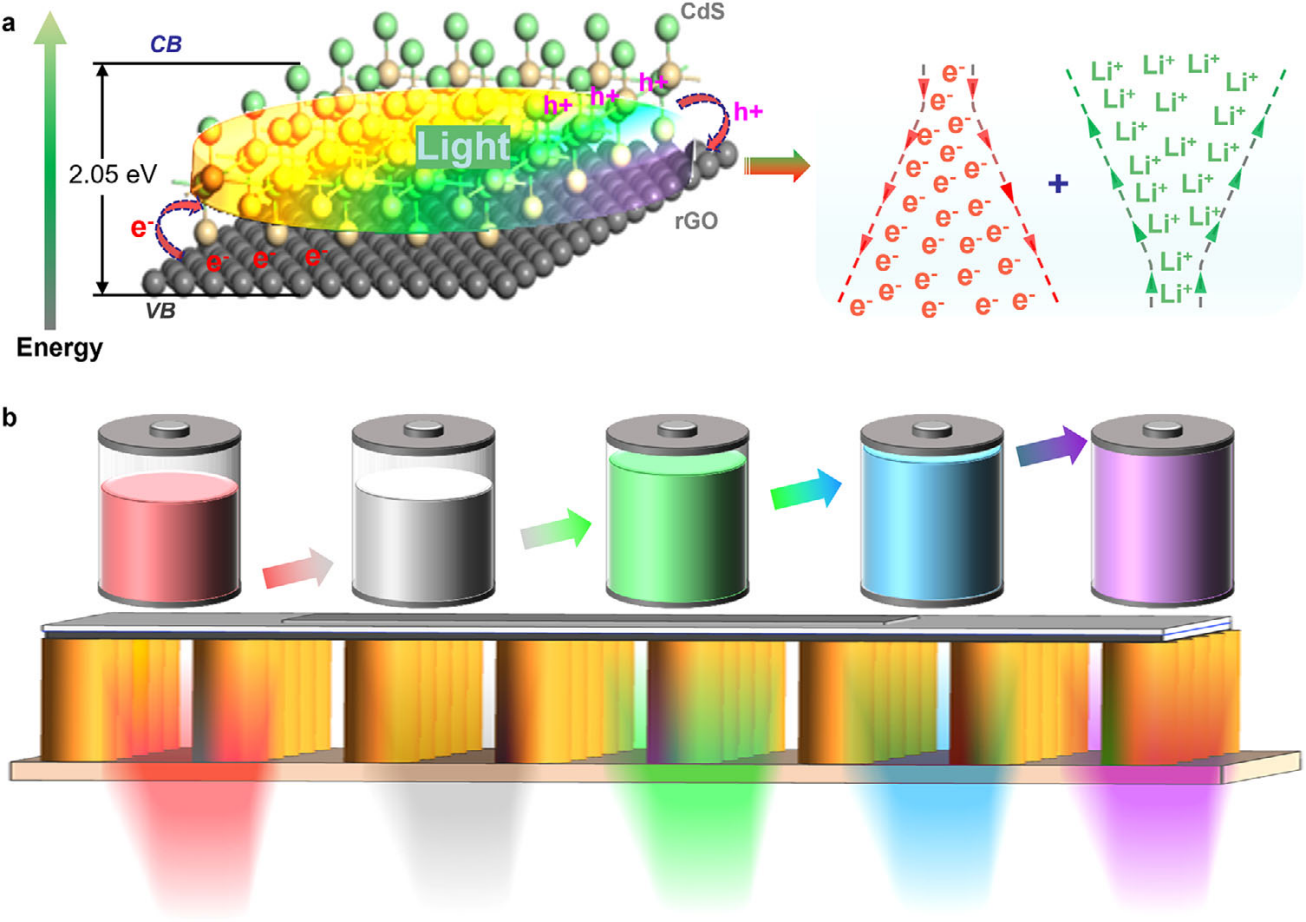
a) Influence of the CdS/rGO heterojunction on electronic transfer and Li‐ion diffusion. b) Schematic illustration of capacities and light sources for half‐cell.

**Figure 2:**
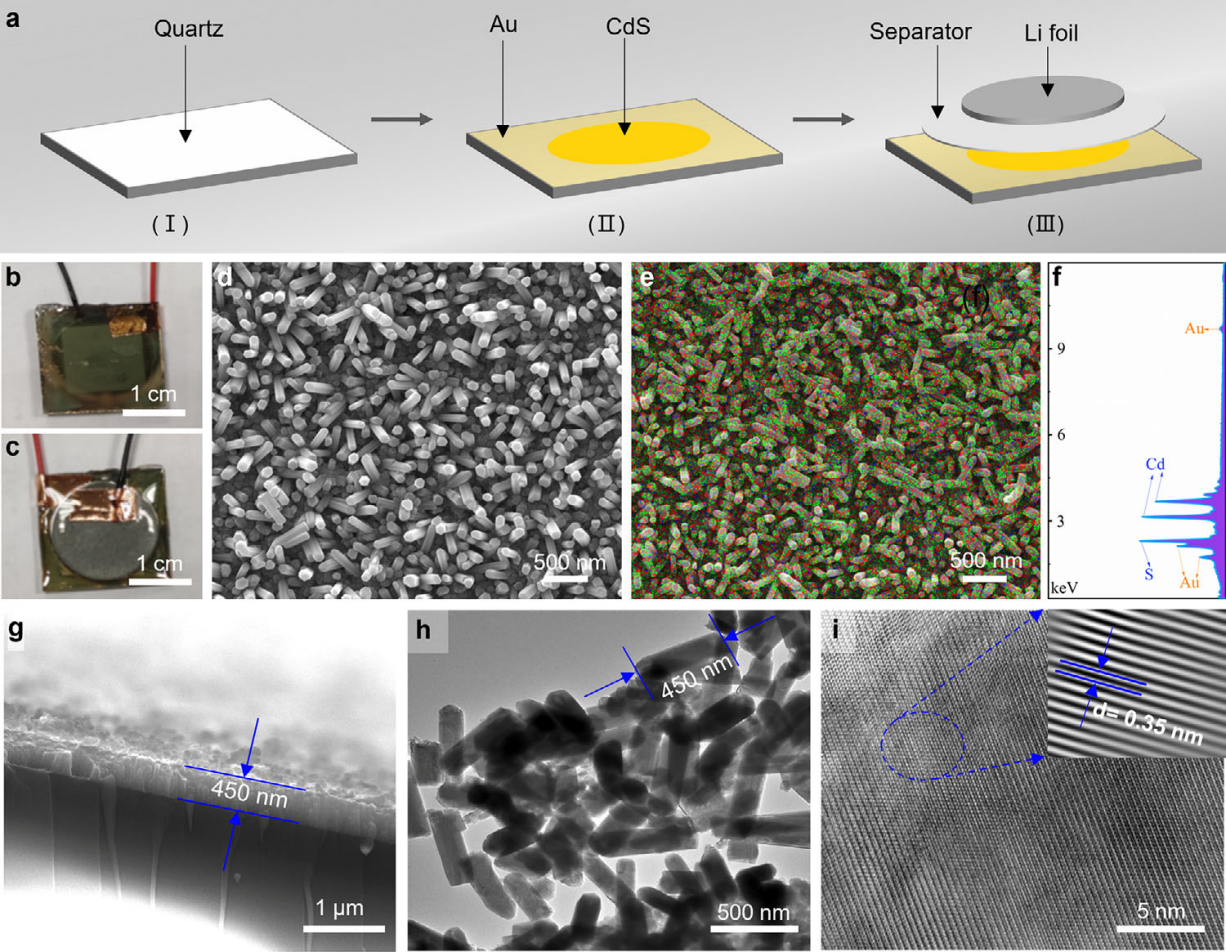
a) Light‐regulated battery pack consisting of a transparent quartz window, CdS anode, rGO electronic enhancement layer, and Li‐foil counter electrode. b,c) Optical images of the front face and back face of the light‐regulated LIB, respectively. d) SEM image of CdS nanorod arrays. e) Mixed element mapping images of Cd, S, and Au. f) EDS spectra of the CdS nanorod arrays. g) Cross‐section SEM image of the CdS nanoarrays on the quarte substrate. h) TEM image of CdS nanorod arrays. i) High‐resolution TEM image of CdS nanorod arrays, insert is the corrected lattice.

The schematic illustration in **Figure** **1**a depicts the action mechanism of the CdS/rGO heterojunction in the broadband light‐modulated anode design. The electronic band alignment, as shown in Figure S1 (Supporting Information), reveals that the formation of the heterojunction suppresses the electron transfer from rGO to CdS, while facilitating the transport of excess holes from CdS to rGO under illumination, attributed to their different work functions. Specifically, CdS/rGO heterojunction perform well in electron‐hole separation and transport under illumination and charge–discharge cycles. Photogenerated electron‐hole pairs are formed by CdS absorbing photons. Due to CdS's higher conduction band position than rGO's Fermi level, photogenerated electrons swiftly inject into rGO and are efficiently carried along its conductive network, preventing electron‐hole recombination. While photogenerated holes stay in CdS and react with electrolyte redox species, recombination probability decreases. During discharge, rGO transports electrons from the electrode material to the external circuit, while CdS holes go through its semiconductor network to the current collector, participating in electrochemical processes. Electrons from the external power source enter rGO and fill CdS holes to restore active materials during charging. The synergy of light irradiation and charge‐discharge operations boosts the CdS/rGO heterojunction's photoelectric conversion efficiency and electrochemical performance, giving it excellent cycling stability and ion storage. Consequently, an inherent potential field is established at the interface, promoting electronic transportation and ionic diffusion.^[^ ^14^ ^]^ This phenomenon can optimize the conductivity, impedance, and overall Li‐storage performance. Additionally, the absorption wavelength of the CdS/rGO composite, as depicted in Figure S2 (Supporting Information), influences the number of photo‐generated carriers, thereby modulating the intensity of the built‐in potential field, which subsequently affects the electronic transportation and ionic diffusion capabilities. As a result, the capacity and impedance can be modulated by varying the wavelengths (Figure 1b). A “transparent” light‐accepting battery device is developed and fabricated to realize this concept, as illustrated in **Figure** **2**a. Initially, CdS nanorod arrays are directly grown on a quartz window (Figure 2a I), which is subsequently coated with 5 nm‐thick gold nanoparticles using electron beam evaporation (Figure 2a II). This structure serves as the working electrode for constructing the light‐modulated anode. The cell components include a highly transparent and conductive quartz window, working electrode, separator, Li‐foil counter electrode, and gasket (Figure 2a III). The components are sealed in a glove box using polydimethylsiloxane (PDMS) and UV resin, ensuring excellent cell sealability and translucency.

The optical images of the light‐modulated LIB are presented in Figure 2b,c. The front side image (Figure 2b) exhibits a solid and smooth appearance, indicating the excellent cohesiveness of polydimethylsiloxane (PDMS) and the fast‐curing property of UV resin. On the back side (Figure 2c), the separator and copper conducting filler are visible, highlighting the high transparency of the constructed device. In addition, the crystal structure of the as‐prepared CdS nanorod arrays are determined by X‐ray diffraction (XRD), and the corresponding pattern is shown in Figure S3 (Supporting Information). The diffraction peaks of CdS nanorods can be distinguished and corresponds to *P63mc* space group of the hexagonal crystal system (JCPDS File No. 77–2306). Compared with the two weak peaks at 27 and 28° of CdS, the sharp diffraction peak (002) at 26.2° directs highly oriented CdS nanorods growth along the CdS crystalline *c*‐axis. The Au film coated on the quartz is also evidently detected with a pair of peaks at 39 and 44°, which can be assigned to the cubic phase Au. Raman spectra are employed to confirm the presence of carbon in the CdS/rGO composite (Figure S4, Supporting Information). The two characteristic peaks observed at 1363 and 1583 cm^−1^ can be attributed to the disorder‐induced feature (D band) and the E_2g_ patterns of graphite carbon (G band), respectively. X‐ray photoelectron spectroscopy (XPS) spectra are further utilized to elucidate the structural characteristics of the CdS nanorods (Figure S5, Supporting Information). The binding energies of Cd 3d_3/2_ and 3d_5/2_ are determined to be 411.9 and 405.1 eV, respectively, consistent with previous reports. Additionally, the peaks at 162.9, 161.8, and 161.7 eV can be assigned to the 2p_3/2_, 2p_1/2_, and 2p states of sulfur, respectively.^[^ ^15^ ^]^ These results confirm the purity of the CdS nanorod arrays without any oxidized states of sulfur and cadmium atoms presented in the cell. The CdS nanorod arrays have been grown on the quartz substrate and exhibit a dense morphology with uniform distribution (Figure 2d). CdS nanoarray with a well‐defined hexagonal shape and an average diameter of ≈150 nm (Figure S6, Supporting Information) can be observed. Notably, there are distinct gaps between each nanorod, which facilitate light transmission within the CdS arrays. The Cd, S, and Au are distinctly confirmed by mixed element mapping images (Figure 2e) and EDS spectra (Figure 2f). Following the spin coating and thermo‐reduction processes, the reduced graphene oxide (rGO) is uniformly distributed on the CdS nanorods (Figure S7, Supporting Information). The length of the CdS nanorods is determined to be ≈450 nm from the cross‐sectional SEM in Figure 2g and transmission electron microscopy (TEM) image in Figure 2h, indicating a minor aspect ratio that contributes to more effective photoresponse. The measured d‐spacing of the lattice is ≈0.35 nm, corresponding to the (001) plane of hexagonal wurtzite CdS (Figure 2i).^[^ ^16^ ^]^ This directional growth of single crystals may positively impact the separation of electrons and holes.^[^ ^15^ ^]^

### 2.2 Phase Transformation and Work Mechanism of the Light‐Modulated Anode

**Figure 3:**
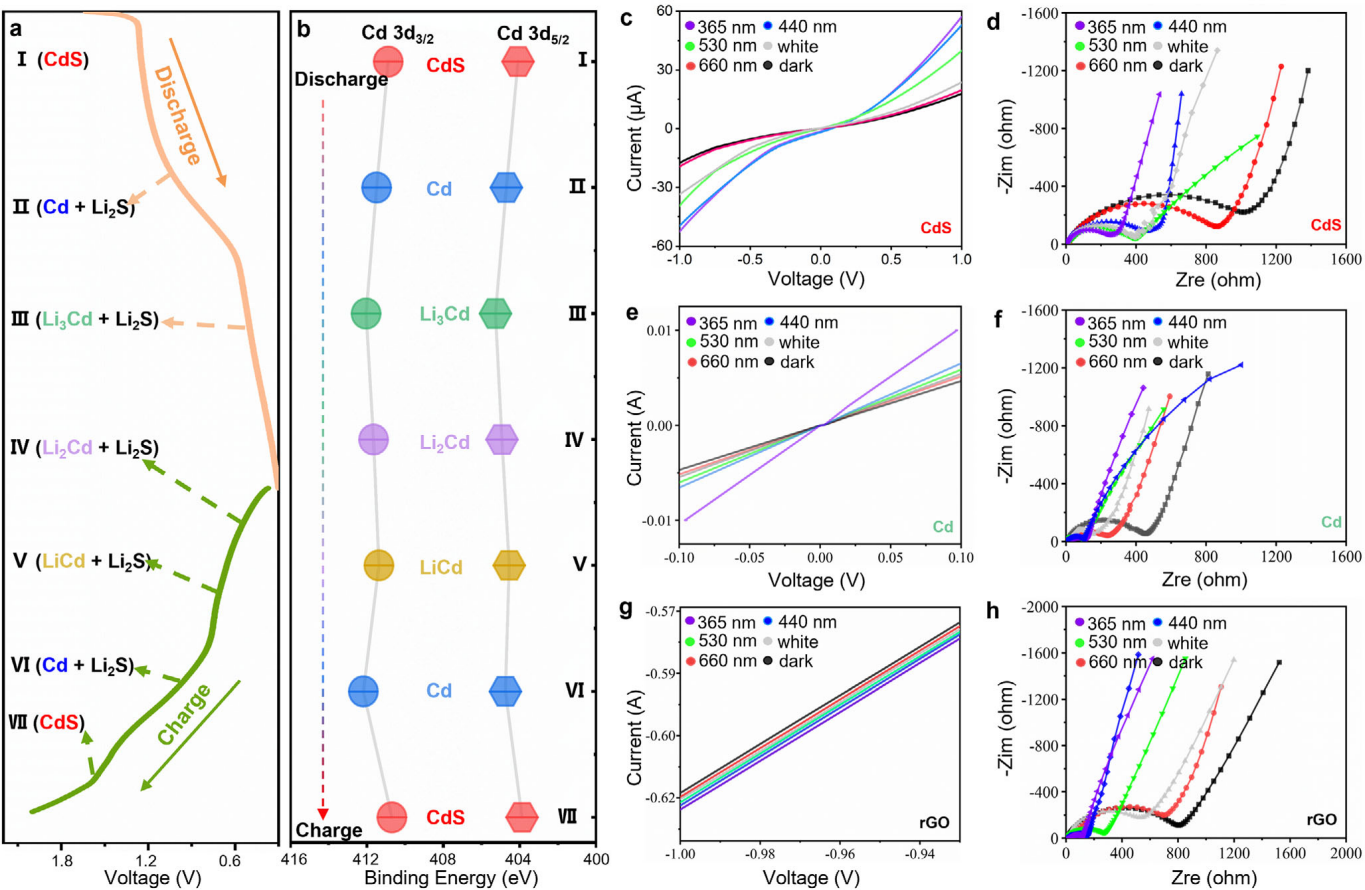
a) Schematic diagram illustrating the seven states of the CdS/rGO electrode during lithiation/delithiation processes. b) Evolution of binding energies in Cd 3d_3/2_ and 3d_5/2_ orbitals in the CdS anode during the entire discharge/charge cycle. c) *I–V* curves of the as‐fabricated CdS/rGO photodetector device under varying wavelength light illumination (2 mW cm^−2^ intensity) and in darkness. d) Nyquist plots depicting the impedance spectra under different wavelength light illuminations (2 mW cm^−2^ intensity) and in darkness for the initial state. e) *I–V* curves of the Cd metal device under different wavelength light illuminations (2 mW cm^−2^ intensity) and in darkness. f) Nyquist plots showing impedance spectra under different wavelength light illuminations (2 mW cm^−2^ intensity) and in darkness for a state discharged to 0.7 V. g) *I–V* curves of the graphene device under different wavelength light illuminations (2 mW cm^−2^ intensity) and in darkness. h) Nyquist plots illustrating impedance spectra under different wavelength light illuminations (2 mW cm^−2^ intensity) and in darkness for a state discharged to 0.20 V.

Cyclic voltammetry (CV) and charge/discharge profiles are employed to investigate the phase transformation of the CdS/rGO working electrode, as illustrated in Figure S8 (Supporting Information). The redox behavior and plateau characteristics suggest the presence of intermediate phases such as Cd, LiCd, Li_2_Cd, and Li_3_Cd (**Figure** **3**a). To validate this assumption, the valence changes of the Cd atom are examined using a rapid sample preparation technique for XPS. Figure 3b depicts the general evolution of binding energies for Cd 3d_5/2_ and 3d_3/2_ orbitals in the CdS anode during the charge/discharge process, including seven stages (initial state I, discharging stage II at 1.05 V and stage III at 0.5 V, charging stage IV at 0.47 V, stage V at 0.69 V, stage VI at 0.77 V and stage VII at 1.59 V).

At stage I, during the initial discharge, the Cd atom in a high valence state, mainly regarded as CdS, exhibits elevated binding energies due to the loss of two electrons from the 4s orbital and the strong attraction of the nucleus toward the remaining shell electrons. Upon discharge to 1.05 V (stage II), the Cd 3d_5/2_ and 3d_3/2_ peaks are observed at 404.53 and 411.30 eV, respectively, which can be attributed to Cd (Figure S9a, Supporting Information). The higher binding energies suggest easier electron access to the atomic shell, indicating the presence of elementary cadmium in stage I based on binding energies and previous reports.^[^ ^17^ ^]^ Subsequently, upon further discharge to 0.50 V (stage III), the elementary form of cadmium shows lower binding energy due to the fully occupied 3d and 4s orbitals (3d^10^4s^2^). The binding energies of Cd 3d_5/2_ and 3d_3/2_ are shifted to lower values of 403.96 and 410.73 eV, respectively (Figure S9b, Supporting Information). This shift implies that the shells are filled with electrons, which can be attributed to a Li_3_Cd phase, a cadmium alloy. Notably, a weak peak at 398.80 eV corresponds to the N 1s peak, possibly resulting from the reaction between the lithium‐cadmium alloy and nitrides in the ambient air. The N 1s peak is also observed during the subsequent charge process involving Li_2_Cd and LiCd alloys. Extra electrons in the outer shell result in even lower binding energies, as this necessitates overcoming a higher potential energy barrier.^[^ ^7^, ^18^ ^]^

When charging back to 0.47 V (stage IV) (Figure S9c, Supporting Information), the binding energies of Cd 3d_5/2_ and 3d_3/2_ peaks increase to 404.35 and 411.07 eV, respectively, indicating the transformation from Li_3_Cd to Li_2_Cd. Upon further charging to 0.69 V (stage V), the binding energies of Cd 3d_5/2_ and 3d_3/2_ continue to rise to 404.63 and 411.45 eV, respectively (Figure S9d, Supporting Information), signifying the formation of LiCd. Upon reaching a charge of 0.77 V (stage VI), the binding energies shift to 403.82 and 411.25 eV (Figure S9e, Supporting Information), similar to the values observed in Figure S9b (Supporting Information), which can be attributed to the Cd phase. Finally, upon charging to 1.59 V (stage VII), the binding energies (Figure S9f, Supporting Information) resemble those of Cd^2+^ in Figure S5 (Supporting Information), confirming the formation of the CdS phase (stage VI). Consequently, the following equations can represent the overall discharge/charge process of the CdS/rGO working electrode.

Discharge process,

(1) $$\mathrm{CdS}+2Li^{+}+2e^{-}=\mathrm{Cd}+Li_{2}S\text{…}.1.05\;V$$

(2) $$\mathrm{Cd}+3Li^{+}+3e^{-}=Li_{3}\mathrm{Cd}\text{…}\text{…}\text{…}\text{…}.0.50\;V$$

Charge process,

(3) $$Li_{3}\mathrm{Cd}-e^{-}=Li_{2}\mathrm{Cd}+Li^{+}\text{…}\text{…}\text{…}\text{…}0.47\;V$$

(4) $$Li_{2}\mathrm{Cd}-e^{-}=\mathrm{LiCd}+Li^{+}\text{…}\text{…}\text{…}\text{…}..0.69\;V$$

(5) $$\mathrm{LiCd}-e^{-}=\mathrm{Cd}+Li^{+}\text{…}\text{…}\text{…}\text{…}\text{…}..0.77\;V$$

(6) $$\mathrm{Cd}+S^{2-}-2e^{-}=\mathrm{CdS}\text{…}\text{…}\text{…}\text{…}\text{…}\text{…}.1.59\;V$$

The *I–V* curves and electrochemical impedance spectroscopy (EIS) are employed to elucidate the fundamental working mechanism of LM‐LIB comprehensively. According to reaction types during the charge/discharge, the light‐modulated process of the CdS/rGO working electrode can be divided into three parts: conversion, alloying, and dealloying. During the conversion process, the CdS can be transformed into an intermediate Cd metal phase (stages I to II), and the photoelectric nature primarily arises from CdS.^[^ ^19^ ^]^ Due to CdS/rGO heterojunction, a photodetector device is constructed to investigate the electronic transport property. By varying light illumination from ultraviolet to visible with a constant intensity of 2 mW cm^−2^, the photocurrent versus voltage (*I–V*) curves exhibit distinct characteristics (Figure 3c). Notably, the CdS/rGO photodetector device exhibits pronounced photovoltaic behavior, with photovoltaic parameters such as open‐circuit voltage (V*_oc_*) and short‐circuit current density (I*_sc_*) increasing with higher photon energy (Figure S10, Supporting Information). Simultaneously, the ion diffusivity of the light‐modulated LIB is assessed using EIS (Figure 3d). The experimental data are fitted utilizing simplified contact models based on Randles' equivalent circuit (Figure S11, Supporting Information). The results demonstrate that the membrane resistance (R*_f_*) values of the CdS/rGO electrode vary with different light illuminations, ranging from 14.61 to 3.92 Ω. Similarly, the charge‐transfer resistance (R*_ct_*) values exhibit variation, ranging from 1156 to 289.6 Ω. The observed changes in R*_f_* and R*_ct_* values substantiate the fundamental theory of light‐modulated LIB. Furthermore, the Li‐ion diffusion coefficients are examined under different light conditions, revealing an inverse relationship with the “*σ*” values (Note S1, Supporting Information). Specifically, the “*σ*” values range from 15.45 to 77.54, corresponding to light illuminations at 365, 440, 530 nm, white light, 660 nm, and dark, with an intensity of 2 mW cm^−2^ (Figure S12, Supporting Information). These findings indicate that light illumination effectively modulates the conductivity and ion diffusivity of the CdS/rGO working electrode during the initial phase.

Second, during the alloying process (stages II to III), the intermediate Cd can be alloyed into Li_3_Cd, and the Cd/rGO would dominate the light‐modulated process due to the absorption peak of Cd metal can be found at 800 nm (Figure S13, Supporting Information). To verify the photocurrent, the Cd/rGO photodetector is measured under different light and dark states (Figure 3e). Under light irradiation, the electrode resistance decreases significantly, showing the desired wide spectral response and wavelength characteristics. In addition, the EIS spectra from the intermediate states are evaluated to illustrate the ion diffusivity with light irradiation (Figure 3f). The R*_f_* and R*_ct_* values are 3.85, 4.37, 5.02, 5.43, 6.54, 10.67 Ω, and 99.3, 110.9, 111.5, 151.1, 235.3, 436.9 Ω at the illumination of 365, 440, 530 nm, white, 660 nm and dark with an intensity of 2 mW cm^−2^, respectively. After linear fitting, the “*σ”* values are calculated to be 31.82, 37.34, 40.99, 56.19, 115.41, and 187.11, respectively (Figure S14, Supporting Information). The results indicate that light could well modulate the conductivity and ion diffusivity during the alloying process.

As for the dealloying process, Li_3_Cd can be transformed into Li_2_Cd and LiCd. However, the chemical properties of the resulting Li*_x_* Cd alloy are unstable, making it challenging to capture the photoelectric performance of Li*_x_* Cd effectively. The band gaps of Li*_x_* Cd exhibit interrelation in the vicinity of the Fermi level, suggesting the absence of photoresponse (Figure S15, Supporting Information). Therefore, as a reasonable approximation, the desirable photoresponse performance of the anode can be attributed to rGO.^[^ ^20^ ^]^ To further analyze the photoresponse characteristics, the partially enlarged *I–V* curve of the rGO photodetector is presented in Figure 3g. Additionally, Figure S16 (Supporting Information) illustrates the independent *I–V* curves of rGO under different illumination conditions, owing to the significant dark current exhibited by graphene. Despite rGO's suitability as an electron carrier, the rGO photodetector demonstrates an available photoresponse performance. The evaluation of ion diffusivity is also conducted in this stage, as shown in Figure 3h. The calculated values of R*_f_* and R*_ct_* are as follows: 4.67, 6.68, 9.23, 11.26, 14.94, 18.27 Ω, and 109.2, 170.4, 229.8, 510.2, 612.5, and 795.8 Ω at illuminations of 365, 440, 530 nm, white light, 660 nm, and in the dark, respectively, with an intensity of 2 mW cm^−2^. Furthermore, the corresponding “*σ*” values, indicative of the ion diffusivity, are determined to be 43.78, 50.39, 64.15, 67.99, 75.59, and 87.02 under the same illumination conditions mentioned above (Figure S17, Supporting Information). In summary, the results as mentioned above provide compelling evidence that the conversion, alloying, and dealloying processes of the Cd/rGO composite material could be efficiently light‐modulated, as demonstrated by the significant decrease in resistance and the modulation of ionic diffusivity.

### 2.3 Li‐Storage Properties of the Light‐Modulated Anode

The electrochemical performance of the CdS/rGO electrode is initially assessed through galvanostatic discharge/charge measurements without illumination. The CdS/rGO electrode demonstrates a first discharge capacity of 732.8 mAh g^−1^ and a charge capacity of 378.1 mAh g^−1^ at a current density of 0.2 A g^−1^, resulting in an initial coulombic efficiency of 51.6% (Figure S18a, Supporting Information). Remarkably, the charge/discharge plateaus are consistent with the observed redox peaks, notably a discharge plateau at 1.05/0.5 V and a charge plateau at 0.75/1.53 V, respectively. These observations indicate the exceptional structural stability and electrochemical reversibility of the CdS/rGO electrode.^[^ ^21^ ^]^ After 20 cycles, the capacity stabilizes, and by the 200th cycle, it reaches 275 mAh g^−1^, maintaining 94% of the capacity observed at the 20th cycle. (Figure S18b, Supporting Information). The improvement in Li‐storage performance observed in the CdS/rGO electrode can be attributed to the synergistic enhancement of electron and Li‐ion transport kinetics facilitated by the internal electric field within the CdS/rGO heterojunction.^[^ ^22^ ^]^ Besides, the CdS nanorod arrays, combined with the high electronic conductivity of the gold film, provide preferential pathways for rapid electron transport during the charge/discharge process.^[^ ^23^ ^]^ As a comparison, we collected CdS nanorod powder synthesized by hydrothermal method and CdS nanorod arrays without rGO coating to serve as contrast electrodes. Both the electrodes exhibit inadequate Li‐storage properties, with capacities of merely 94.1 and 123.9 mAh g^−1^, respectively (Figure S19, Supporting Information). The rate performance of the CdS/rGO electrode is evaluated after 20 cycles at 0.2 A g^−1^, as shown in Figure S20 (Supporting Information). Remarkably, there was only a slight decrease in the discharge capacity even when the current density was incrementally increased from 0.2 to 15 A g^−1^. Specifically, the discharge capacities measured at current densities of 0.2, 0.5, 1.0, 2.0, 4.0, 8.0, and 15 A g^−1^ were 285, 261, 228, 175, 135, 121, and 111 mAh g^−1^, respectively, demonstrating the exceptional rate capability of the CdS/rGO electrode.

**Figure 4:**
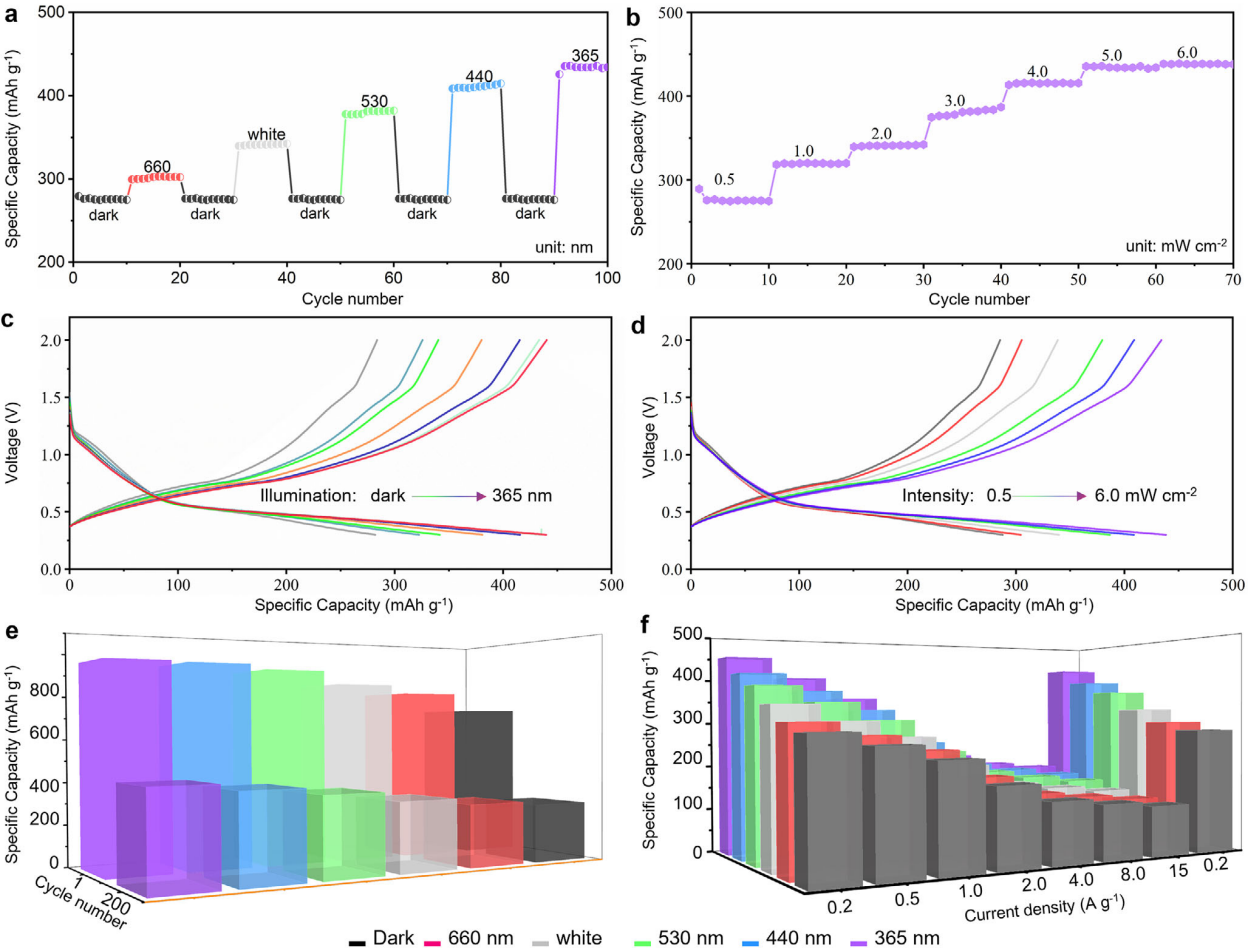
a) Cycling performance of CdS/rGO electrode with different light sources at a constant light intensity of 5.0 mW cm^−2^ (current density is 0.2 A g^−1^). b) Charge/discharge profiles of CdS/rGO electrode with different light sources at a constant light intensity of 5.0 mW cm^−2^ (current density is 0.2 A g^−1^). c) Cycling performance of CdS/rGO electrode with different light intensities from 0.5 to 6.0 mW cm^−2^ at a constant wavelength of 365 nm (current density is 0.2 A g^−1^). d) Charge/discharge profiles of CdS/rGO electrode with different light intensities from 0.5 to 6.0 mW cm^−2^ at a constant wavelength of 365 nm (current density is 0.2 A g^−1^). e) First and 200^th^ capacity of CdS/rGO electrode with different light source at a constant light intensity of 5.0 mW cm^−2^ (current density is 0.2 A g^−1^). f) Rate performance histogram of CdS/rGO electrode with different light source at a constant light intensity of 5.0 mW cm^−2^.

The broadband light‐modulated performance of the CdS/rGO electrode is comprehensively assessed under various light illuminations and intensities (**Figure** **4**). Without any illumination, the CdS/rGO electrode exhibits an initial capacity of 275 mAh g^−1^. However, when subjected to intermittent illuminations at wavelengths of 660 nm, visible light, 530, 440, and 365 nm, with a constant intensity of 5 mW cm^−2^, the electrode displays enhanced capacities of 302, 342, 382, 411, and 435 mAh g^−1^, respectively (Figure 4a). Upon removal of the light, the electrode's capacity reverts to its original value of 275 mAh g^−1^. The fluctuating Li‐storage properties observed in the CdS/rGO electrode align with the “On‐Off responses” characteristic of CdS photodetectors,^[^ ^24^ ^]^ consistent with the UV–vis absorption spectrum of the CdS material (Figure S2, Supporting Information). Leveraging the superior light‐modulated property of the CdS/rGO electrode, the maximum Li‐storage performance is evaluated using fixed‐wavelength illumination at 365 nm. The electrode delivers capacities of 275, 320, 341, 381, 415, 434, and 438 mAh g^−1^ at illumination intensities of 0.5, 1.0, 2.0, 3.0, 4.0, 5.0, and 6.0 mW cm^−2^, respectively (Figure 4b). Notably, the obtained capacities are proximate at illumination intensities of 5.0 and 6.0 mW cm^−2^ due to the approaching saturable concentration of photo‐generated carriers in the CdS semiconductor material. Consequently, the resistance of the CdS/rGO electrode remains constant at an illumination intensity of 5 mW cm^−2^, with the capacity showing a subtle upward trend.

The charge/discharge curves for the CdS/rGO electrode under different wavelengths and light intensities are shown in Figure 4c,d. These curves are quite similar to those seen in Figure S18a (Supporting Information), and the voltage plateaus align with the discussions. This suggests that the cadmium sulfide electrode retains stable electrochemical characteristics when exposed to light. Figure 4e illustrates the prolonged cycling performance of the CdS/rGO electrode under various light sources, maintaining a constant light intensity of 5.0 mW cm^−2^ and a current density of 0.2 A g^−1^. Without illumination, the CdS/rGO electrode has a capacity of 275 mAh g^−1^ after 200 cycles. Under continuous illumination at 660 nm, white light, 530, 440, and 365 nm, the capacities after 200 cycles are 293, 323, 368, 399, and 430 mAh g^−1^, respectively. A detailed analysis of the long‐term cycling performance reveals that both illumination and wavelength significantly influence the Li‐storage properties of the CdS/rGO electrode (Figure S21, Supporting Information). Figure 4f presents the variation pattern of the rate performance for the CdS/rGO electrode under different light sources with the same light intensity of 5.0 mW cm^−2^. The CdS/rGO electrode demonstrates excellent rate performance, attributable to its well‐designed hierarchical structure. Notably, the rate capability with 365 nm illumination showcased more stable Li‐storage performance at higher current densities, delivering a notable capacity of 176 mAh g^−1^ at 15 A g^−1^, outperforming the capacity of the electrode without illumination (111 mAh g^−1^). The quantum yield and the number of electron‐hole recombinations based on the capacity fluctuation of the CdS/rGO electrode were evaluated at different wavelengths, as shown in Tables S1 and S2 (Supporting Information). The results demonstrate that as photon energy rises (wavelength diminishes), the ratio of recombined electron‐hole pairs progressively increases, indicating that although the quantity of photogenerated carriers markedly escalates at shorter wavelengths, the fraction of electrons effectively engaged in charge transport diminishes. This behavior is presumably due to the increased photon energy at shorter wavelengths, which produces more excitons at the interface; yet, some of these electron‐hole pairs quickly recombine without being successfully harnessed.

These findings collectively highlight the sensitivity of the Li‐storage property and electron transport capability of the CdS/rGO electrode, which can be modulated by varying wavelengths and intensities of light. Under UV light of less than 365 nm, high‐energy photons efficiently excite the photogenerated electron‐hole pairs in CdS, creating a robust built‐in electric field at the interface. This enables the segregation of photogenerated carriers and enhances electron transfer to rGO, markedly decreasing interfacial resistance and improving Li^+^ diffusion efficiency. At 440 and 530 nm, when photon energy diminishes, the generation efficiency of photogenerated carriers drops, impairing electron‐hole pair separation and marginally elevating interfacial resistance, although electronic transport capability remains essentially stable. As the wavelength extends to 660 nm, the subsequent decline in photon energy results in a substantial decrease in photogenerated electron‐hole pairs, hence constraining electron transport, elevating interfacial resistance, and markedly diminishing the Li⁺ diffusion rate. In summary, by adjusting the intensity of the intrinsic electric field across various wavelengths, the electron transport and Li^+^ diffusion capabilities of the CdS/rGO heterojunction can be efficiently managed, enabling precise control over electrochemical performance.

### 2.4 Li‐Storage Properties of the Light‐Modulated Anode

**Figure 5:**
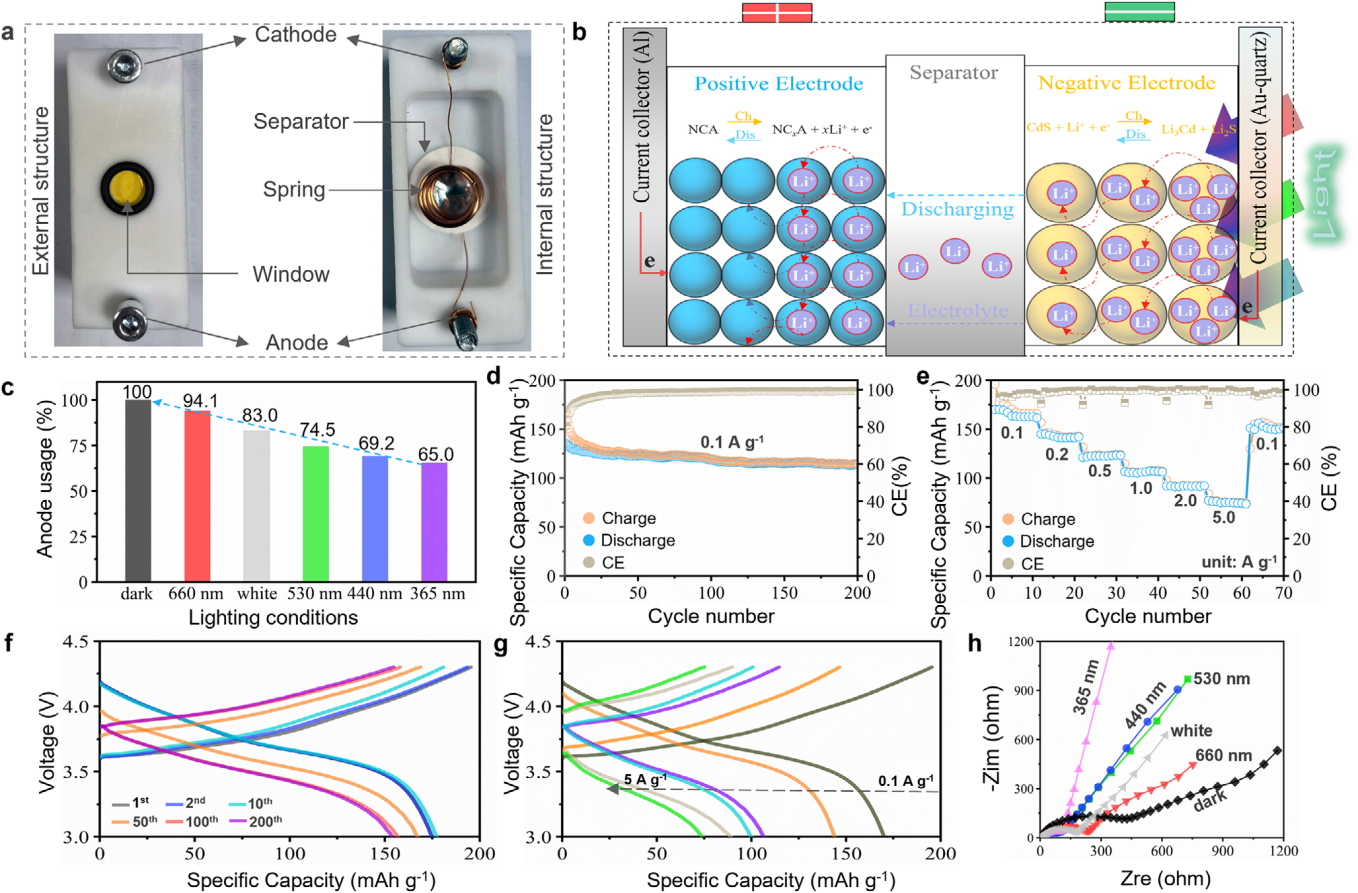
a) Structure diagram of the full‐cell with NCA cathode versus CdS/rGO anode couple. b) Working mechanism of the full‐cell. c) Anode usages matching with CdS/rGO anode under different lighting conditions. d) Cycling performance of the full‐cell with light intensity of 6.0 mW cm^−2^ at a constant wavelength of 365 nm (current density is 0.1 A g^−1^). e) Rate performance of the full‐cell with light intensity of 6.0 mW cm^−2^ at a constant wavelength of 365 nm (current density is from 0.1 to 5 A g^−1^). f) Charge/discharge profiles of the full‐cell with light intensity of 6.0 mW cm^−2^ at a constant wavelength of 365 nm (current density is 0.1 A g^−1^). g) Charge/discharge profiles of the full‐cell with light intensity of 6.0 mW cm^−2^ at a constant wavelength of 365 nm (current density is from 0.1 to 5 A g^−1^). h) EIS of the full‐cell with light intensity of 6.0 mW cm^−2^ at different illumination.

The electrochemical performance of CdS/rGO is assessed in a full‐cell configuration employing Al_2_O_3_‐coated LiNi_0.8_Co_0.15_Zn_0.05_O_2_ (Al‐NCZ) as the cathode material. The choice of Al‐NCZ as the cathode material is grounded in its outstanding Li‐storage performance in half‐cells (theoretical capacity of 279 mAh g^−1^, 1 C = 0.279 A g^−1^).^[^ ^25^ ^]^ This renders it a promising candidate for LIBs due to its high voltage plateaus, favorable performance, and cost‐effectiveness. The preparation details of the Al‐NCZ cathode are reported in our previous work. **Figure** **5**a illustrates the assembly of the structure diagram of the full‐cell. It comprises the cathode, anode, separator, transparent window, and spring. A sealant and a sealing ring are employed to ensure adequate sealing performance. The full‐cell working mechanism and reactions are demonstrated in Figure 5b.^[^ ^26^ ^]^ In the charging process, the Li‐ions continuously transport from the Al‐NCZ cathode to CdS/rGO anode, and the reaction will be reversed in the discharge process. It is worth noting that the N/P ratio of the full‐cell is 1.05.^[^ ^27^ ^]^ In addition, benefiting from the variable capacities of CdS/rGO anode, we also evaluate the cathode usage (Figure 5c). When designing a full‐cell for efficient operation, the capacity of the anode typically exceeds that of the cathode by ≈5%. Therefore, with the cathode capacity fixed and after normalization, it is found that the consumption of the anode material in the absence of light is 35% more than under ultraviolet light exposure. The full‐cell exhibits a capacity of 125 mAh g^−1^ at 0.1 A g^−1^ after 200 cycles (Figure 5d), under a light intensity of 6.0 mW cm^−2^ at a consistent wavelength of 365 nm. The rate performance test, Figure 5e, shows the full‐cell can deliver high reversible capacities of 165, 142, 123, 106, 92, and 76 mAh g^−1^ at 0.1, 0.2, 0.5, 1.0, 2.0, and 5.0 A g^−1^, respectively, under a light intensity of 6.0 mW cm^−2^ at a consistent wavelength of 365 nm. The galvanostatic and rate charge/discharge profiles (Figure 5f,g) exhibit a similar voltage plateau, consistent with the redox reactions occurring at the Al‐NCZ cathode. In addition, the full‐cell EIS data have been checked at different illuminations with a constant intensity (Figure 5h), indicating that there is the lowest R*_ct_* value at 365 nm with the most rapid Li‐ion diffusion. In contrast, the opposite scenarios are observed in the absence of illumination. Thus, it suggests that the CdS/rGO anode can be applied successfully in the full‐cell with its variable capacities under illumination. This promising performance exhibits significant potential for enhancing the energy storage capabilities for next‐generation batteries and paving the way for the widespread adoption of renewable energy sources.

## 3 Conclusion

In this study, we have conducted a comprehensive investigation to demonstrate the feasibility of in situ LM‐LIBs across UV to visible light spectrum. By introducing the photoconductive CdS semiconductor with high capacity into the anodic design, a heterojunction consisting of n‐type CdS and p‐type rGO has been successfully constructed, which not only realizes in situ light‐modulated capabilities throughout the charge/discharge processes but also significantly improves the Li‐ion kinetics. CdS/rGO anodes can deliver a capacity of 430 mAh g^−1^ under illumination of 365 nm, nearly twice as high as that under dark conditions (275 mAh g^−1^) at 0.2 A g^−1^ after 200 cycles. Additionally, the impedance values are adjusted swiftly from 1025 to 261 Ω under different illuminated conditions. Furthermore, in a full‐cell configuration where the cathode material quantity is fixed, the anode material needed under conditions of illumination is reduced by 35% compared to the requirement in the absence of light, thereby effectively enhancing the energy density of LIB. Owing to the unique properties of CdS semiconductor materials and the incorporation of light‐modulated capabilities, this approach opens up new avenues for advancing the performance and functionality of LIBs across diverse applications.

## 4 Experimental Section

### Preparation of CdS Nanorod Arrays

According to the methodology outlined in our previous research,^[^ ^28^ ^]^ the fabrication process of CdS nanorod arrays on an ultrathin silica glass (USG) substrate involved several carefully executed steps. First, the USG substrate was meticulously prepared by subjecting it to a thorough ultrasonic cleaning procedure. The cleaning process involved sequential immersion of the substrate in high‐purity acetone (99.8%, Sigma–Aldrich), ethanol (99.8%, Sigma–Aldrich), and deionized water. This rigorous cleaning ensured the removal of any organic impurities or contaminants, thus providing a pristine substrate surface for subsequent processing steps. Following the cleaning process, a 5 nm gold film was deposited onto the USG substrate using a high vacuum electron beam evaporation technique. This deposition step served as a catalyst for the subsequent growth of CdS nanorod arrays. Subsequently, the prepared USG substrate with the gold film was carefully placed inside an autoclave. The autoclave contained a precisely formulated solution composed of cadmium nitrate (99.8%, Sigma–Aldrich), thiourea (99.8%, Sigma–Aldrich), and glutathione (99.8%, Sigma–Aldrich). The molar ratio of each component in the solution was ≈: 1: 0.6, with a specific focus on maintaining a concentration of cadmium ions at 15 mmol·L^−1^. Finally, the autoclave was heated to a controlled temperature of 200 °C and maintained at this temperature for a duration of 8 h. This thermal treatment facilitated the growth and formation of CdS nanorod arrays on the USG substrate, resulting in the desired nanostructured morphology.

### Fabrication of CdS/rGO Heterojunction

A suspension of graphene oxide (GO) with a concentration of 1 mg·mL^−1^ (99.8%, Sigma–Aldrich) was applied onto the top surface of the CdS nanorod arrays using a spinning coating technique. This process ensured a uniform and controlled deposition of GO onto the nanorod arrays. To remove any residual solvent, the nano heterojunction structure was subjected to a vacuum drying process in a specially designed oven set at a temperature of 110 °C. Following the vacuum drying step, the nano heterojunction structure underwent further heat treatment in an atmosphere comprising argon (Ar) gas with a composition of 90% and hydrogen (H_2_) gas with a composition of 10%. This specific gas mixture was introduced to create a reducing environment necessary for the reduction of graphene oxide (GO). The heat treatment process took place at a temperature of 450 °C and lasted for a duration of 6 h. The elevated temperature and the reducing gas environment facilitated the reduction of GO, leading to the formation of rGO and promoting the formation of a CdS/rGO heterojunction.^[^ ^29^ ^]^

### Rechargeable LIB Assembly

The as‐prepared CdS/rGO heterojunction sample served as the working electrode in the experimental setup. The cells were assembled using lithium foil as the counter electrode. The electrolyte consisted of a 1 m LiPF_6_ solution in a mixture of ethylene carbonate and dimethyl carbonate (EC/DMC) in a 1: 1 volume ratio. Celgard 3501, a commercially available separator from Celgard, LLC Corp., USA, was utilized to separate the electrodes within the cell. To ensure proper sealing of the device, UV curing glue was applied and cured using a UV light source with a wavelength of 365 nm and an intensity of 5 mW cm^−2^. All cell assembly procedures were performed inside a glove box with extremely low water and oxygen content, both maintained below 0.1 ppm, to prevent any undesirable reactions. The experiments were conducted at room temperature. To regulate the operation of the rechargeable lithium‐ion battery devices based on light, a series of light‐emitting diodes (Thorlabs) with different central wavelengths were utilized. The intensity of illumination could be adjusted by varying the voltage source of the function generator and employing an optical attenuator.

### CdS/rGO Photodetector Fabrication

First, an etched fluorine‐doped tin oxide (FTO) conducting glass was utilized as the substrate for the growth of the CdS nanorod array. The growth of CdS nanorods on the FTO substrate was achieved using a hydrothermal method, which involved the controlled reaction of precursor solutions in an aqueous environment. The hydrothermal process enabled the formation of the CdS nanorod arrays on the FTO substrate. To establish the desired heterojunction, a layer of GO film was spin‐coated onto the CdS nanorod arrays. The spin‐coating technique ensured uniform coverage and adherence of the GO film onto the nanorod array surface. Subsequently, the GO film underwent a heat treatment process to induce rGO. The heat treatment played a crucial role in enhancing the electrical conductivity and stability of the resulting rGO film. To complete the photodetector structure, a silver paste was applied onto the FTO conducting glass, serving as the bottom electrode. Additionally, a carbon paste was deposited onto the rGO film, functioning as the top electrode. The silver pastes and carbon paste served as electrical contacts for the photodetector, enabling the measurement of photocurrent generated by incident light. The fabricated CdS nanorod array photodetector was characterized and its performance was evaluated under different illumination conditions. The photodetector's active area was ≈1 cm^2^, determined by the intersection area between the CdS nanorod arrays and rGO film. Photodetector performance tests were conducted using light sources of various wavelengths and intensities. The experimental results were recorded using a digital camera, and further analysis was performed to assess the device's responsiveness and sensitivity to different light stimuli.

### Material Characterization

Morphological characterizations of the CdS nanorods and CdS/rGO heterojunction were performed by scanning electron microscopy (SEM, NOVA 450, FEI) and transmission electron microscopy (TEM, G30 FEI). The crystalline structures of the as‐prepared materials were characterized by X‐ray diffraction (XRD, Shimadzu XRD‐6000). Raman spectra of the products were collected on a Thermo scientific FT‐Raman spectrometer (FRA 106/s) using an Nd‐line laser source with an excitation wavelength of 532 nm. The valence states analysis of the CdS NRs was performed with an X‐ray electron spectrometer (XPS, AXIS‐ULTRA DLD‐600 W).

### Semiconductor Properties Testing

All the *I–V* characteristics of the device were recorded by a semiconductor analysis system (Keithley 4200).

### Electrochemical Testing

The light‐regulation rechargeable lithium‐ion battery devices were placed in camera obscura and a series of light emitting diode (Thorlabs) with different central wavelengths as the light sources. The luminous intensities were adjusted by the voltage and optical attenuator. The Galvanostatic Charge–discharge test was conducted on a LAND cycler (CT 2001, Wuhan Kingnuo Electronic Co., China). Cyclic voltammetry (CV) and electrochemical impedance spectroscopy (EIS) measurements were carried out with the coin cells using a CHI 760D electrochemical workstation (ChenHua Instruments Co., China).

## Conflict of Interest

The authors declare no conflict of interest.

## Supporting information



Supporting Information

## Data Availability

The data that support the findings of this study are available from the corresponding author upon reasonable request.
