# Supplementary material for: In Situ Light‐Modulation of Capacity and Impedance in Lithium‐Ion Batteries
Source: Adv Sci (Weinh). 2025 Jun 5;12(32):e03340. doi: 10.1002/advs.202503340 (PMC12407316; doi:10.1002/advs.202503340)
Supplement: Supplementary file 1 — Supporting Information [file ADVS-12-e03340-s001.docx]

**Supporting Information**

***In*-situ Light-Modulation of Capacity and Impedance in Lithium-Ion Batteries**

*Hong Yin,^1,2,*^ Xiangxiang Yu,^3^ Yucan Zhu,^1^ Zhaohui Hou,^1^* *Joao Cunha^2^, Zhenxing Liang,^4,^* Zhipeng Yu,^2,^**

^1^Key Laboratory of Hunan Province for Advanced Carbon-based Functional Materials, Hunan Institute of Science and Technology, Yueyang, 414006, China

^2^International Iberian Nanotechnology Laboratory (INL), Braga, 4715-330, Portugal

^3^School of Integrated Circuits, Huazhong University of Science and Technology, Wuhan, 430074, China

^4^Guangdong Provincial Key Laboratory of Fuel Cell Technology, School of Chemistry and Chemical Engineering, South China University of Technology, Guangzhou, 510641 China

*Corresponding author

Email: 2017507027@hust.edu.cn (H. Yin); zliang@scut.edu.cn (Z. Liang); zhipeng.yu@inl. int (Z. Yu)

Supplementary Figures


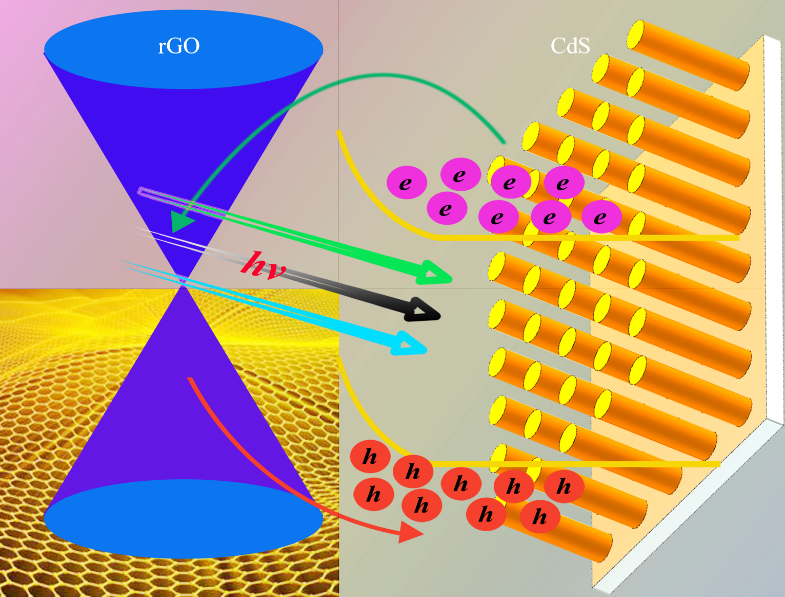


**Figure S1.** Energy band diagram of CdS/rGO heterojunction.


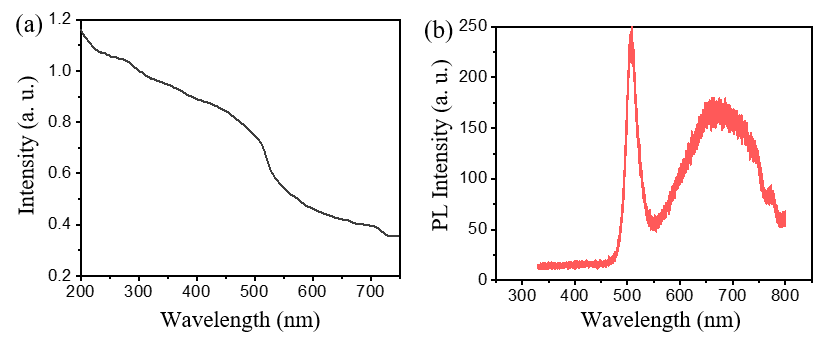


**Figure S2.** UV-vis spectrum (a) and fluorescence spectrum (b) of CdS/rGO.


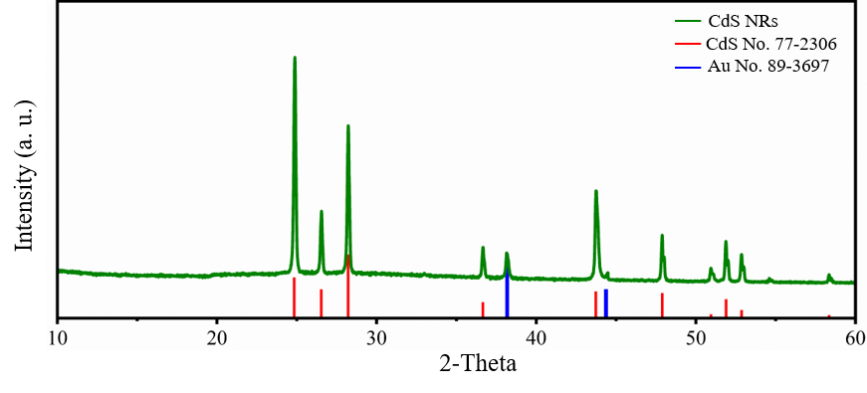


**Figure S3.** XRD pattern of CdS nanorod arrays.


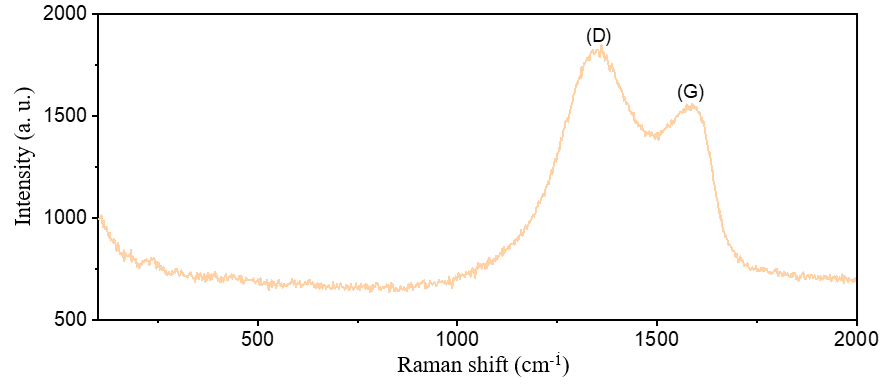


**Figure S4.** Raman spectrum of the CdS/rGO composite.


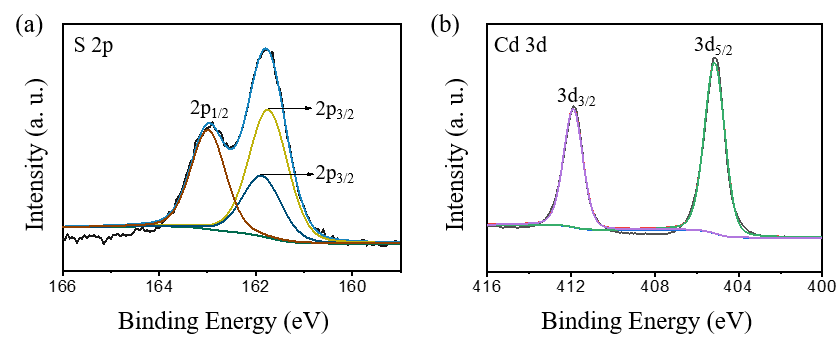


**Figure S5.** XPS spectra of S 2p and Cd 3d, respectively.


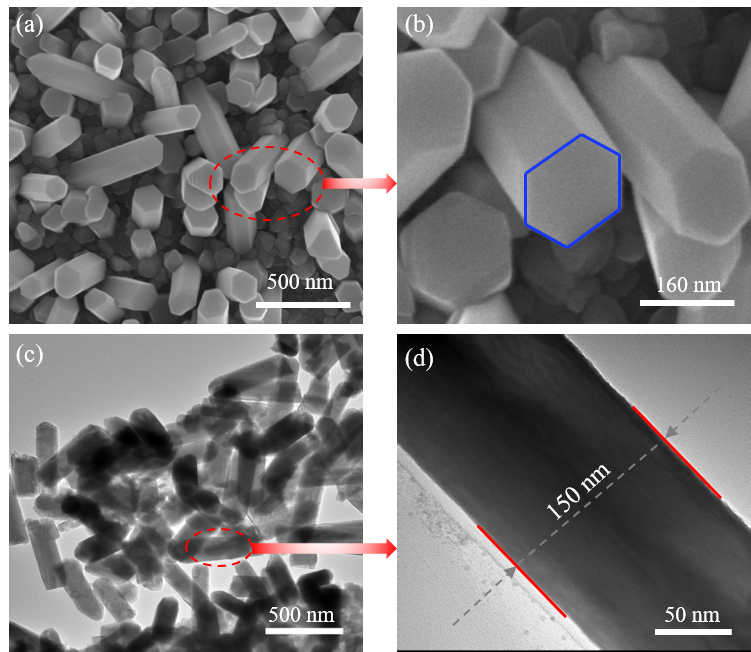


**Figure S6.** (a) SEM image of CdS nanorods. (b) Magnification image of the select CdS nanorod (a). (c) TEM image of CdS nanorods. (d) Magnification image of the select CdS nanorod (c).


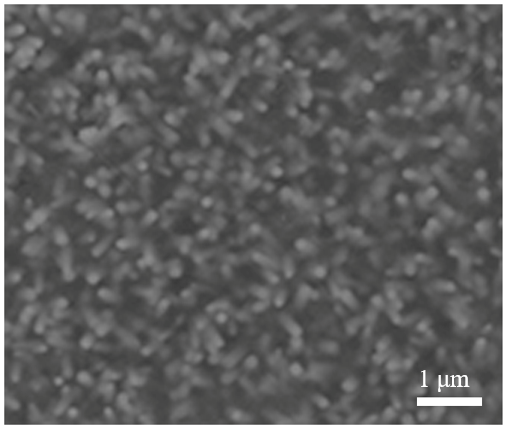


**Figure S7.** SEM image of rGO coated on CdS nanorod arrays.


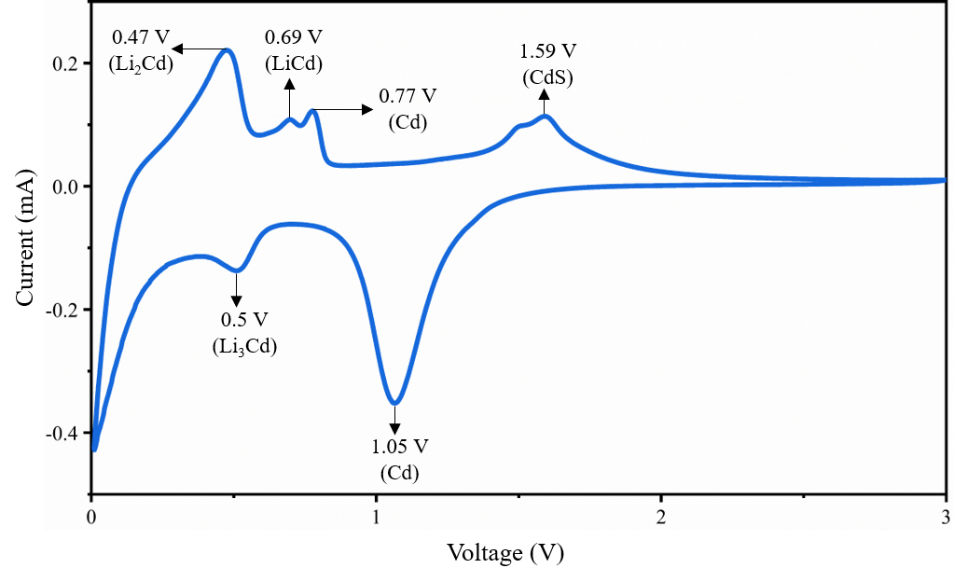


**Figure S8.** CV curve of the DLCM anode from 0.01 V to 3.0 V with a scan rate of 0.1 mV s^–1^ after two pre-cycles.


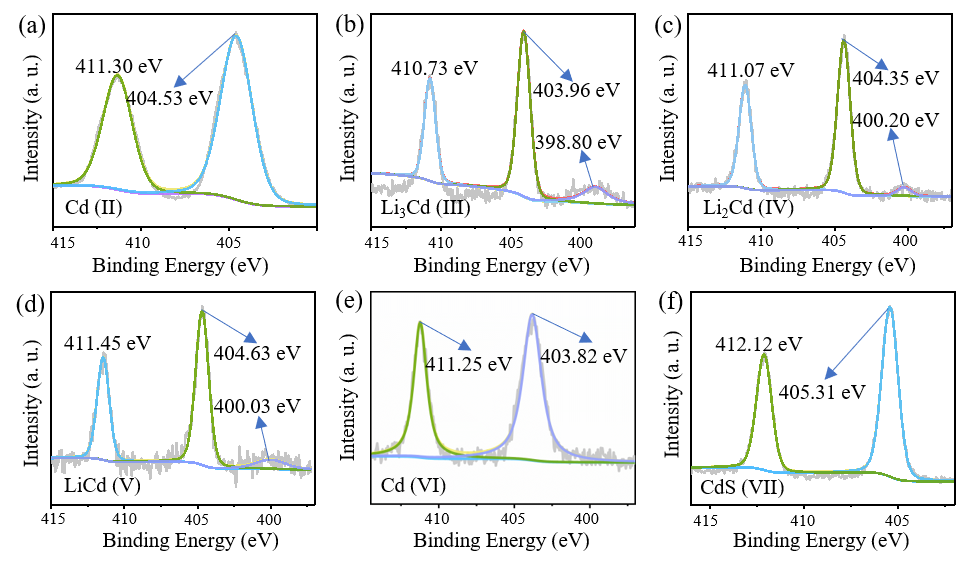


**Figure S9.** (a-b) Binding energies of CdS anode at the defined discharge voltage of 1.05 and 0.50 V during the discharge process, respectively. (c-f) Binding energies of DLCM anode at the defined charge voltage of 0.47 V, 0.69 V, 0.77 V and 1.59 V during the discharge process, respectively.


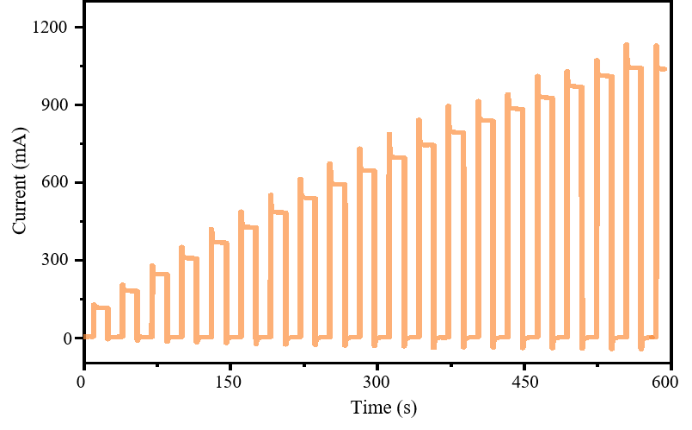


**Figure S10.** Time-resolved photoresponse of the CdS/rGO device under UV illumination with variable intensities from dark to 9 mw cm^–2^ at zero bias voltage.


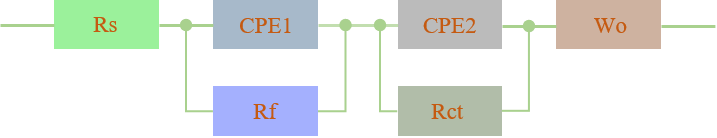


**Figure S11.** Equivalent circuit for the CdS/rGO electrode.


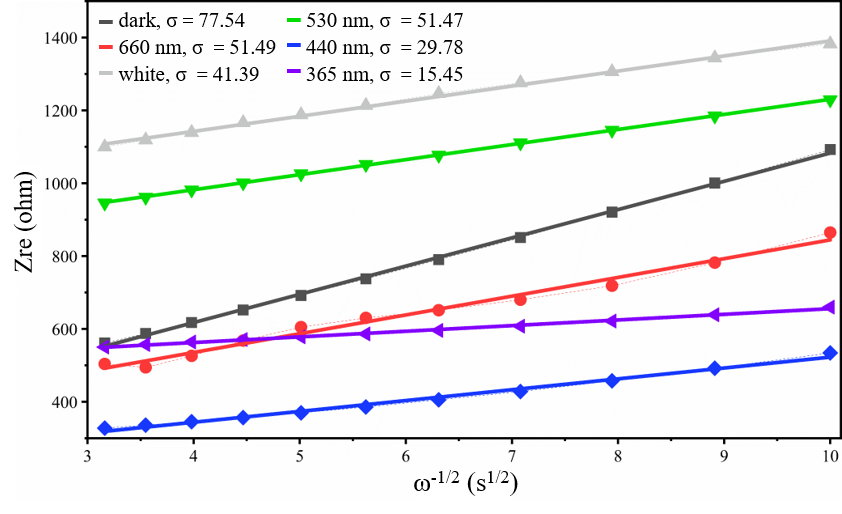


**Figure S12.** Real part of the complex impedance versus ω^–1/2^ at open circuit voltage for the initial state.


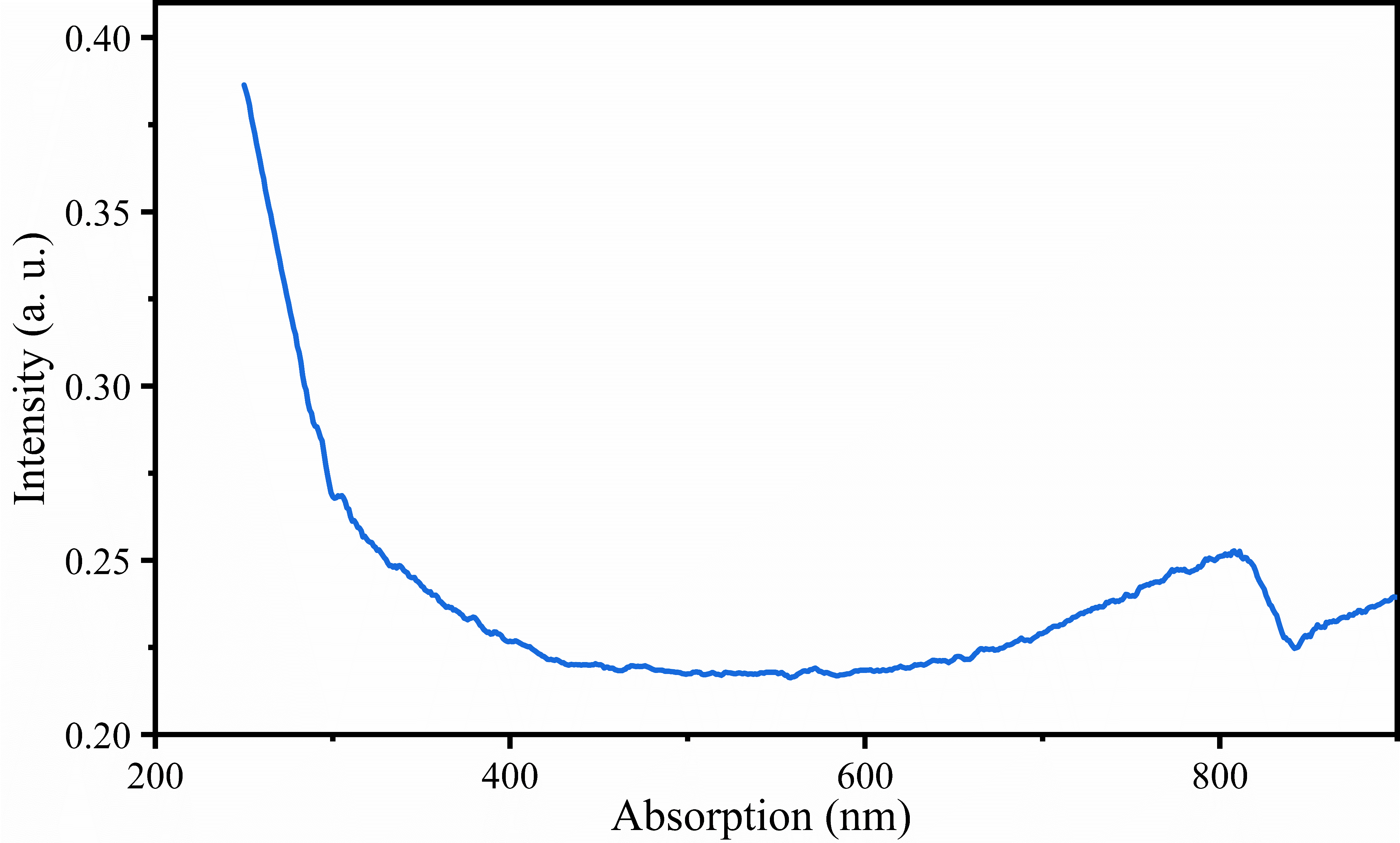


**Figure S13.** UV-vis absorption of Cd nano arrays.


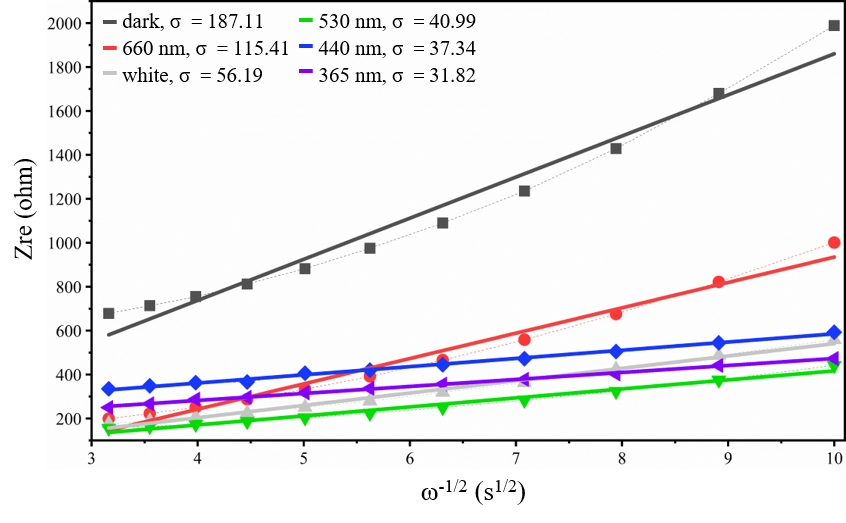


**Figure S14.** Real part of the complex impedance versus ω^–1/2^ at open circuit voltage for the intermediate state.


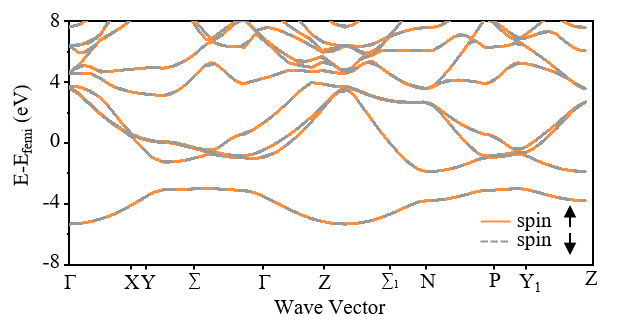


**Figure S15.** Fermi level of Li_3_Cd by DFT simulation.


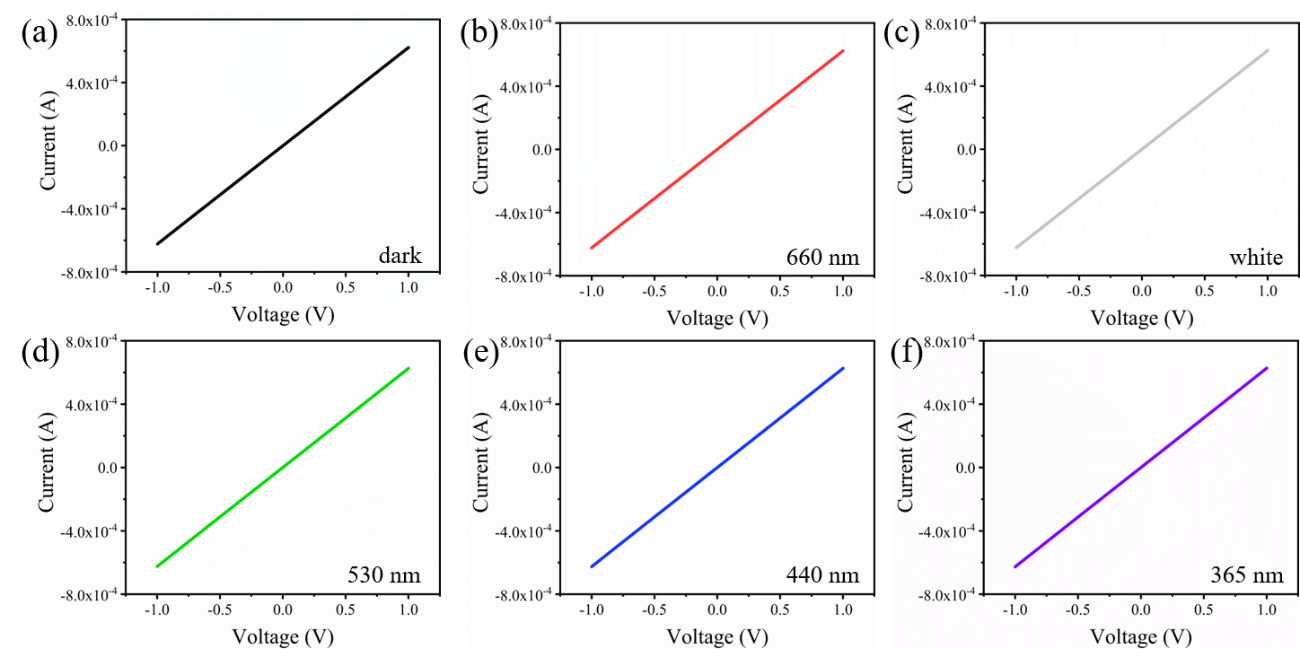


**Figure S16.** Independent I-V curves of rGO at different illumination.


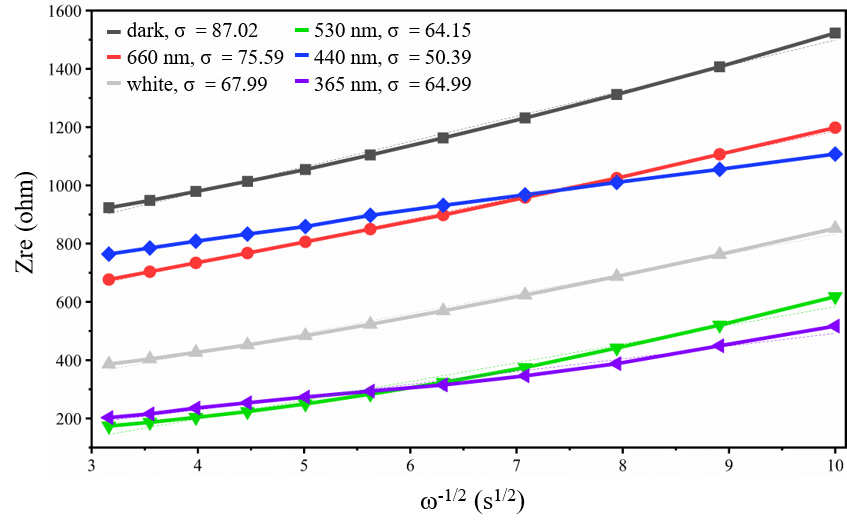


**Figure S17.** Real part of the complex impedance versus ω-1/2 at open circuit voltage for the final state.


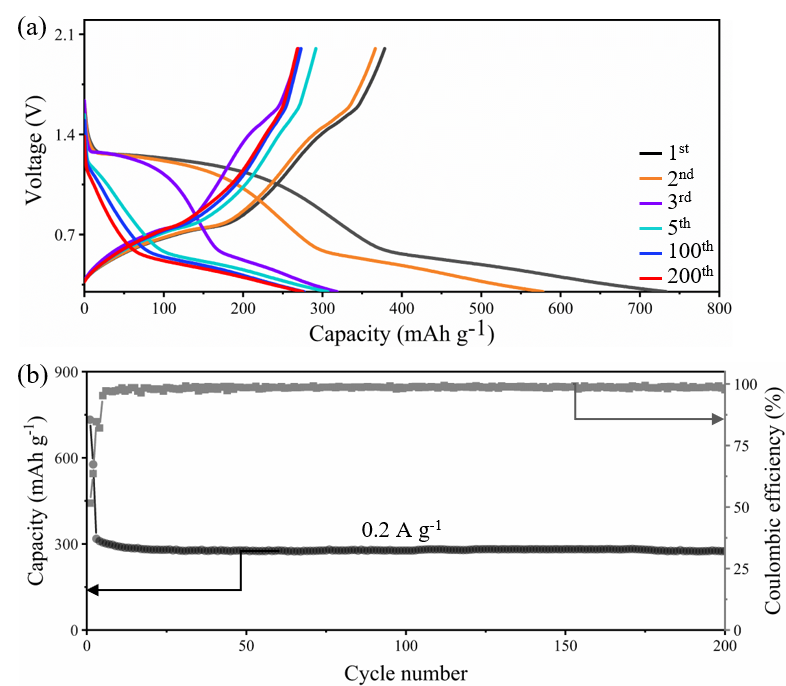


**Figure S18.** (a) Charge/discharge profiles of the DLCM anode between 0.3 V and 2.0 V at the 1^st^, 2^nd^, 100^th^ and 200^th^ cycles with a current density of 0.2 A g^–1^. (b) Cycling performance of the DLCM anode at a current density of 0.2 A g^–1^.


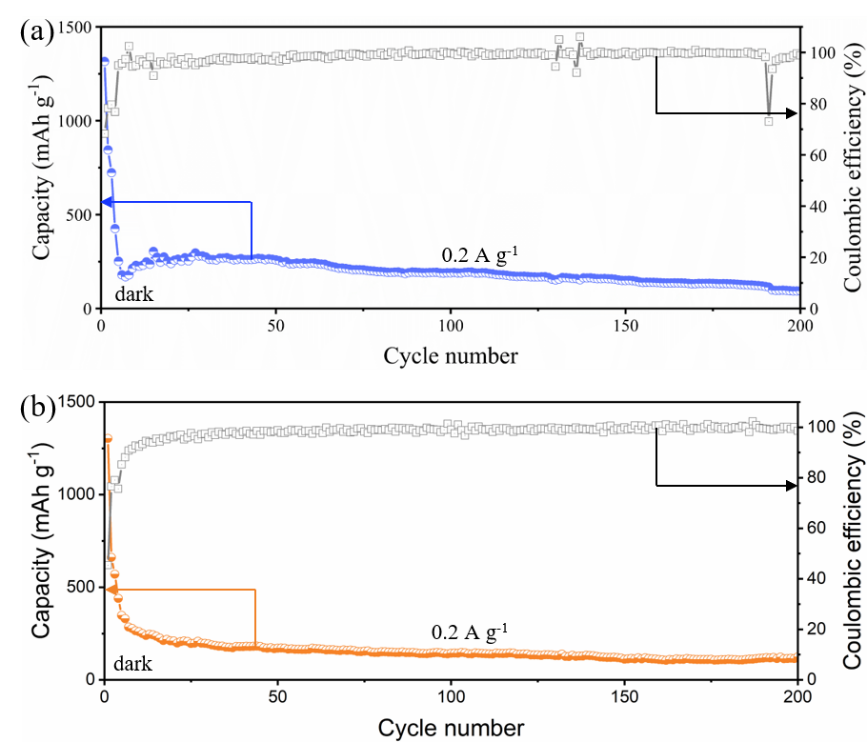


**Figure S19.** Cycling performance of (a) CdS nanorod powder and (b) CdS nanorod arrays, respectively.





**Figure S20.** Rate capability of the DLCM anode at various current densities from 0.2 A g^–1^ to 15 A g^–1^.


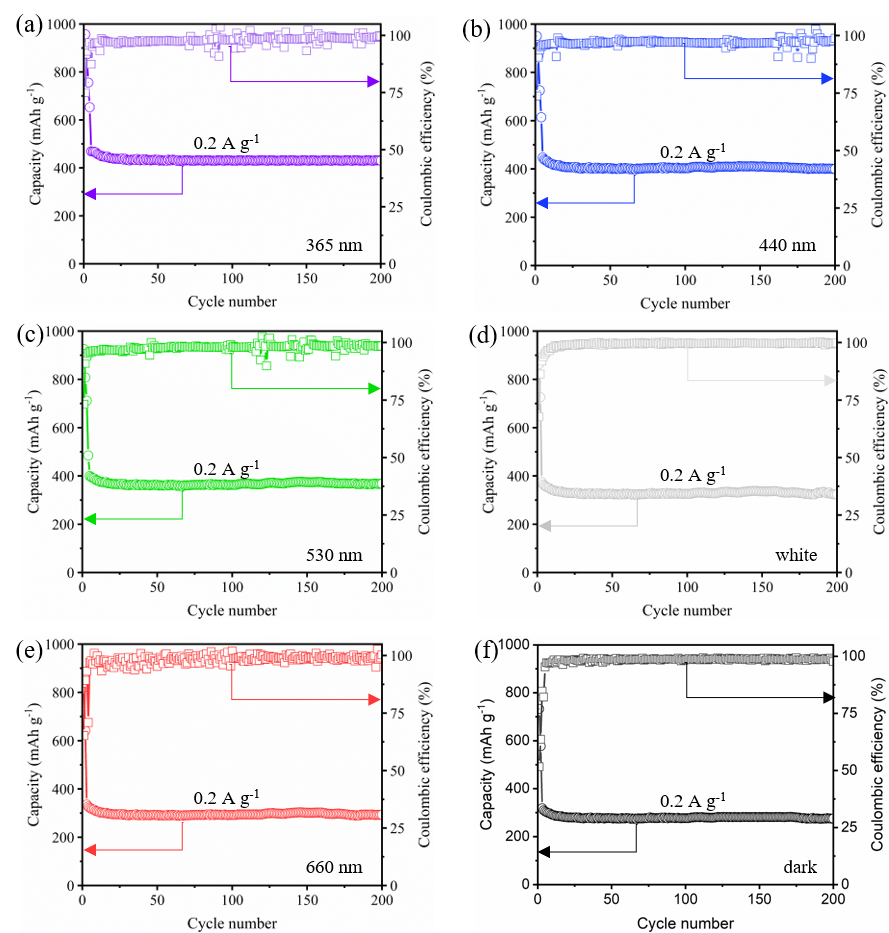


**Figure S21.** Cycling performance of the DLCM anode with different light source of (a) 365 nm, (b) 440 nm, (c) 530 nm，(d) white light, (e) 660 nm and (f) without illumination at a constant light intensity of 5.0 mW cm^–2^.

Supplementary Note 1

Explanation of ion diffusion calculations.

Electric conductivity & lithium-ion diffusion coefficient at open circuit state and the real part of the complex impedance versus ω^–1/2^.

D = R^2^T^2^/2A^2^n^4^F^4^C^2^σ^2 ......^ (1)

Z_Re_ = K + σω^−1/2......................^ (2)

where D is the diffusion coefficient (cm^2^ s^–1^), R is the gas constant (8.31 J mol^–1^ K^–1^), T is the absolute temperature (298 K), A is the surface area of the cathode (1 cm^2^), n is the number of electrons transferred in the half-reaction for the redox couple, F is the Faraday constant (96485 C mol^–1^), C is the is the molar concentration of Li ions in CdS, K is a constant, ω is frequency, and σ is the Warburg factor which corresponds to the slope of the curves.^[1]^

Supplementary Note 2

**Quantum yield calculation:**

The quantum yield (Φ) can be calculated using the formula:

Φ = *n_e_*/*n_ph_* (3)

Where *n_e_* = Number of electrons transferred (measured from current and capacity data), *n_ph_* = Number of incident photons (calculated based on light intensity, wavelength, and exposure area)

Determination of *n_ph_*:

The number of incident photons is calculated using:

*n_ph_* = *PAt*/*E_ph_* (4)

Where *P* = Light intensity (5 × 10^–3^ W/cm^2^), *A* = Illuminated area (1 cm^2^), *t* = Illumination time (3600 s, based on the average charging time estimate), *E_ph_* = Energy per photon, given by:

*E_ph_* = *hc/λ* (5)

Where *h* is Planck’s constant (6.626 × 10^−34^ J·s), *c* is the speed of light (3×10^8^ m s), and *λ* is the wavelength.

Determination of *n_e_*

The number of electrons transferred is calculated from the capacity (*Q*) using:

*n_e_* = *QNa*/*F* (6)

Where: *Q* = Charge (C), *Na* = Avogadro’s number, *F* = Faraday’s constant (96485 C).

Based on the capacities under illumination at different wavelengths, the number of incident photons, transferred electrons, and quantum yield can be calculated. The results are presented in Table S1.

Table S1 Quantum yield of CdS/rGO electrode under different wavelength illumination.

| Wavelength (nm) | *n_e_* | *n_ph_* | *Φ* (%) |
| --- | --- | --- | --- |
| 660 | 4.04 × 10^18^ | 5.98 × 10^19^ | 6.76 |
| 530 | 2.08 × 10^19^ | 4.81 × 10^19^ | 43.51 |
| 440 | 2.78 × 10^19^ | 3.98 × 10^19^ | 69.88 |
| 360 | 3.48 × 10^19^ | 3.26 × 10^19^ | 106.76 |

**Recombination Fraction (*R*):**

The recombination fraction can be calculated as:

*R* = *1 − 1/Φ* (7)

If *Φ* = 1, then *R* = 0, meaning all carriers are successfully extracted.

If *Φ* > 1, then *R* > 0, indicating that some carriers are recombining.

Calculation of recombination electrons and vacancies: The number of recombined electron-hole pairs can be calculated as:

*n_rec_* = *neR* (8)

Where: *n_rec_* = number of recombined electron-hole pairs, *n_e_* = total number of electrons transferred under illumination.

The results are reported in Table S2, according to the quantum yield value.

Table S2 Electron-hole numbers under different wavelength illumination.

| Wavelength | *R* (100%) | *n_rec_* |
| --- | --- | --- |
| 660 | 85.21% | 3.44×10^20^ |
| 530 | 97.70% | 2.04×10^21^ |
| 440 | 98.57% | 2.75×10^21^ |
| 360 | 99.06% | 3.45×10^21^ |

# References

[1] a) T. Guo, Y. Zhou, Z. Wang, J. Cunha, C. Alves, P. Ferreira, Z. Hou, H. Yin, *Adv. Sci.* **2024**, 11, 2310166; b) Z. Wei, D. K. Singh, K. Helmbrecht, J. Sann, Y. Yusim, J. A. Kieser, C. Glaser, M. Rohnke, A. Groß, J. Janek, *Adv. Energy Mater.* **2023**, 13, 2302525.
